# IMPROVING THE PREDICTION OF FUNCTIONAL RECOVERY IN OLDER ADULTS WITH STROKE IN GERIATRIC REHABILITATION USING AN INERTIAL MEASUREMENT UNIT COMBINED WITH THE UTRECHT SCALE FOR EVALUATION OF REHABILITATION

**DOI:** 10.2340/jrm-cc.v8.43129

**Published:** 2025-05-07

**Authors:** Jules J.M. KRAAIJKAMP, Margot W.M. DE WAAL, Niels H. CHAVANNES, Wilco P. ACHTERBERG, Eléonore F. VAN DAM VAN ISSELT, Michiel PUNT

**Affiliations:** 1 Department of Public Health and Primary Care, Leiden University Medical Center, Leiden, The Netherlands; 2 University Network for the Care sector Zuid-Holland, Leiden University Medical Center, Leiden, The Netherlands; 3 National eHealth Living Lab, Leiden University Medical Centre, Leiden, The Netherlands; 4 Research Group Lifestyle and Health, Utrecht University of Applied Sciences, Utrecht, The Netherlands

**Keywords:** geriatric rehabilitation, balance, Technology Assessment, prediction, accelerometer, stroke

## Abstract

**Background:**

Prediction of functional recovery in older adults recovering from stroke is typically based on observational scales, such as the Utrecht Scale for Evaluation of Rehabilitation (USER). Objectively measuring postural sway using inertial measurement devices (IMU) may complement or improve conventional approaches. The aim of this study was to evaluate whether integrating an IMU with USER data enhances the accuracy of predicting functional recovery at discharge.

**Methods:**

This prospective cohort study included older adults (≥ 65 years) recovering from stroke. Postural sway was assessed using an IMU during 2 different balance conditions and analysed using principal component analysis (PCA). Using 3 different regression models, percentage explained variance was compared to assess predictive performance on functional recovery of USER vs an IMU.

**Results:**

The 71 patients included had a mean age of 78 (SD 7.6) and a median time since stroke of 16 days (IQR 19–60). Of the 71 patients, 12 (16.9%) were unable to perform balance condition 2 due to insufficient balance. Of 35 postural sway features displaying reliability for both balance conditions, 12 were selected for PCA. Incorporation of principal components for both balance conditions in the final model increased the explained variance compared to a model in which only USER-mobility at admission was used to predict delta-USER at discharge (*R*^2^ = 0.61 vs 0.30).

**Conclusions:**

Sitting and standing balance as measured by an IMU improves the prediction of functional recovery at discharge compared to USER alone.

## LAY ABSTRACT

After a stroke, older adults often go through rehabilitation to regain mobility and perform daily tasks. To predict how well someone might recover, healthcare professionals typically use observational scales like the Utrecht Scale for Evaluation of Rehabilitation (USER). This study explores whether using small, wearable devices called inertial measurement units (IMUs) to measure balance while sitting and standing can improve predictions. The study included 71 patients with an average age of 78 years. By combining the USER scale with IMU measurements of balance, we found a improvement in predicting patients’ recovery by the time they were discharged. This approach explained more of the variance in recovery outcomes compared to using the USER scale alone.

With an ageing global population, the number of older adults experiencing stroke is increasing rapidly (1). Older adults who experience stroke often show residual functional or emotional problems, cognitive impairment, and fatigue (2). Geriatric Rehabilitation (GR) is a multidimensional collection of diagnostic and therapeutic interventions that play an important role in aiding older adults recover and regain their independence after stroke. The goal of GR is to optimise functional capacity, promote activity, and maintain functional reserve and social participation in older people with disabling impairments (3).

Predicting functional recovery at the start of GR is important for the organisation and content of a rehabilitation programme, informing and setting patient expectations, and as preparation for the discharge procedure. Studies have determined that age, stroke severity, balance, visual-spatial perception, and independence of functioning on Activities of Daily Living (ADL) on admission are important determinants of functional recovery during GR (4–6). These determinants are conventionally assessed using clinical scales such as the National Institutes of Health Stroke Scale (NIHSS) (7) for stroke severity, Barthel index (BI) (8) for ADL independence, and Berg Balance Scale (BBS) (9) for assessing balance.

A promising multidimensional observational instrument for use during GR is the Utrecht Scale for Evaluation of Rehabilitation (USER) (10). The USER was specifically developed to assess progress during rehabilitation and includes items for mobility, selfcare, and cognitive function (10), combining sufficient clinometric properties of GR (11, 12). Two previous studies have assessed the predictive value of USER: 1 study concluded that the USER effectively predicts physical independence in the general stroke population (13), while another study conducted in GR found that it accurately predicted length of stay and discharge location after GR (14). However, validated clinical observational scales have limitations, mainly due to a dependence on the skill and experience of the assessor for scoring and interpretation (15). Therefore, an objective assessment tool would represent an interesting alternative.

In recent years novel eHealth solutions, such as inertial measurement unit (IMU), have proven their worth in objectively measuring and recording human movement (e.g. body posture and upper and lower extremity movements) (16). Compared with clinical scales, data derived from an IMU generally assess different domains of the International Classification of Function, Disability and Health (ICF) (17). For example, an IMU can assess postural sway (ICF domain: body functions & structures), whereas a clinical scale can assess mobility (ICF domain: activities). A potential added value of an IMU is the ability to complement data obtained with clinical scales, thus integrating data from different ICF domains. This type of data integration not only improves clinical observations and data quality (18, 19), but also generates a unique patient digital phenotype (20), insights from which in turn contribute to improved accuracy of functional recovery prediction. Recent studies have indeed shown that, by measuring postural sway, an IMU can reliably assess sitting and standing balance after stroke (21, 22). While an IMU could potentially improve accuracy, to date IMUs have not been used to complement or improve data obtained with clinical scales.

Using an IMU, in this study we added sitting and standing balance to conventional USER outcomes in order to predict functional recovery. Our aim was to evaluate whether integrating an IMU with USER data enhances the accuracy of predicting functional recovery at discharge in older adults recovering from stroke during GR.

## METHODS

### Design & population

In this prospective cohort study, participants were recruited from 4 GR centres in the Netherlands between January 2020 and December 2022. All participants were older adults (≥ 65 years) and had been diagnosed with stroke. Eligible participants were in the sub-acute phase after stroke, were able to comprehend and sign the informed consent, and were capable of understanding and performing simple tasks. Participants were excluded if they were medically unstable or were unable to sit for at least 1 min without support. All participants gave written informed consent. The study protocol received a waiver of consent from the Utrecht medical ethical review committee (METC number: 20–462/C). Data were collected by a physiotherapist and transferred to the researchers as anonymised data untraceable to any individual person.

### Assessments

Baseline characteristics were assessed during admission and comprised age, sex, body mass index (BMI), time since stroke, type of stroke, and hemiparetic side. The following assessments were registered at admission and discharge: ADL functioning was measured using the BI, which ranges from 0 to 20, with higher scores indicating a better ADL performance (8). The BI has demonstrated excellent reliability and validity properties in stroke population (23). Balance was assessed using the BBS and the Trunk Control Test (TCT). The BBS ranges from, 0 to 56, with higher scores indicating a better balance (9), while TCT ranges from 0 to 100, with higher scores reflecting better trunk control (24). Both assessments have shown good reliability and validity in individuals with stroke (25, 26). Mobility was evaluated using the Functional Ambulation Classification (FAC) and the USER -mobility scale. The FAC categorises mobility from 0 (non-functional walking) to 5 (independent walking outside) (27), and has excellent reliability and moderate validity in stroke populations (28). The USER -mobility scale ranges from 0 to 35, with higher scores indicating a better mobility (10). The clinometric properties of USER were assessed in a previous study, which showed sufficient content validity, internal consistency, interrater reliability, and responsiveness in GR (11, 12). All assessments were standard components of routine care.

The USER is an observational instrument that measures physical (independence in ADL activities, mobility and selfcare) and cognitive function. For the purposes of this study, we used only the ‘mobility’ subscale, which consists of 7 items (sitting, standing, transfers, indoor walking, outdoor walking, climbing stairs, wheelchair use). Each item is scored on a 6-point scale (0–5), reflecting different grades of independence, use of aids, and difficulty.

In addition to the clinical instruments, 2 different balance conditions were measured during the first week of admission, 1 sitting and 1 standing. A balance condition was excluded if a participant was unable to perform the condition. The conditions were arranged based on difficulty and executed in the following order: (*i*) sitting unsupported on a wobble cushion with feet touching the ground and knees at a 90° angle for 60 s, and (*ii*) standing unsupported with feet in self-selected position for 60 s.

**Fig. 1:**
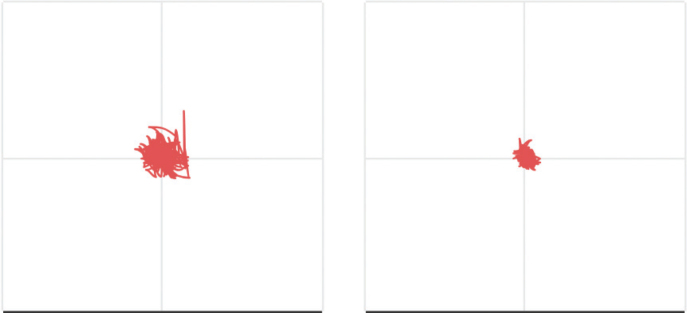
Visualisation of postural sway during balance condition 2. This visualization represents the trajectory of the sensor during measurement in two directions. On the horizontal axis: medio-lateral direction; vertical axis: anterior-posterior direction. A larger surface area presents as greater degree of postural sway, which indicates poorer balance.

Balance conditions were measured using an IMU (manufactured by Aemics B.V. Oldenzaal, The Netherlands), which includes a triaxial accelerometer and gyroscope with a 104x per second sampling rate. The IMU was placed at the estimated height of the participant’s centre of mass; for the seated balance condition, the IMU was placed on the upper back at the T7 level, while for the standing balance condition the IMU was placed on the lower back at the L5/S1 level. The reliability of these balance conditions has been assessed in a previous study and shown to be good to excellent (intraclass correlation coefficient > 0.75) (21). In total, 35 sway features were calculated for every condition, consisting of 21 spatial- temporal features, 8 frequency features, and 6 complexity features, which together describe the quantity, variability, and consistency of movements during the assessment (22). Postural sway is the movement of the centre of mass while in a standing position (29), with increased postural sway generally indicating poor balance (30). A visualisation of postural sway during balance condition 2 is shown in Fig. 1.

### Statistical analysis

Normality of data was tested using the Shapiro-Wilk test. Pearson’s R was used for normally distributed data, which are presented as means with standard deviations (±). Spearman’s rho was used for non-normally distributed data, which are presented as medians with interquartile range (IQR). Outliers for both balance conditions were identified by standardising with Z-scores, with Z-scores ±3 greater than zero removed. Data were analysed using Statistical Package for the Social Sciences; SPSS version 25.0.

### Selection of sway features by principal component analysis

Since all 35 features from the IMU quantify postural sway, they may contain redundant information (21). To address this issue, a PCA was performed to reduce the number of dimensions for the 2 included balance conditions while retaining maximum information (31). Prior to the PCA, the sampling adequacy of all balance conditions was estimated using the Kaiser-Meyer-Olkin measure (KMO). An overall KMO and a per-feature KMO exceeding 0.7 and 0.5 were considered acceptable for analysis (32). To evaluate the robustness and reliability of the principal components, we used test and retest data from a study conducted by Felius et al. (21).

### Predictive modelling

Firstly, principal components of both balance conditions were included as predictors in the regression analysis. Independence of observations was assessed using Durbin-Watson, and variables were assessed for multicollinearity with the variance inflation factor (VIF). Three different regression models were created, with the USER-DELTA (USER-mobility score at discharge minus USER-mobility score at admission) as dependent variable. As independent variables, the first model included the USER-mobility score at admission, the second model included principal components of the balance conditions, and the third model included both the USER-mobility score at admission and the principal components of the balance conditions B1 and B2. The R-squared value and percentage of variance explained (PVE) were compared between the models. Patients with a maximum (optimal) score for USER-mobility at admission were excluded as this precludes evaluation of the functional recovery level (DELTA-USER). Patients who were unable to perform balance condition 2 were also excluded.

To fully understand our main results, we included additional (post-hoc) analyses investigating whether any subgroups would benefit from the addition of IMU sitting and standing balance assessments to the conventional USER assessment. In older adults recovering from stroke, the degree of sitting and standing balance, and therefore mobility, may vary greatly between patients on admission, making some balance assessments very difficult or impossible for some patients while they are too easy for others. We hypothesised that for certain subgroups, based on their level of mobility on admission, sitting and standing balance as measured by an IMU would likely be more accurate in predicting functional recovery after stroke. We therefore defined 3 groups based on their level of mobility independence on admission as measured by the FAC. Group FAC 0 consisted of non-ambulatory participants (FAC score: 0); group FAC 1–3 consisted of participants who needed support during mobilisation (FAC score: 1–3); while group FAC 4–5 included participants who could mobilise independently (FAC score: 4–5). All models were analysed for the entire population, as well as for subgroups defined by the FAC score. Patients who were unable to perform balance condition 2 were excluded.

## RESULTS

A total of 71 patients were included in the study. Patient’s characteristics are described in detail in Table I. The mean age of patients was 78 (SD 7.6), and 38 patients (51%) were male. Regarding type of stroke, 58 (82%) had an ischemic stroke, 11 (15%) a haemorrhagic stroke, and 2 (3%) a subarachnoid stroke.

**Table I:** Patient characteristics at baseline (Mean ±, Median [IQR])

Characteristics	*N* = 71
Age (years)	78±7.6
Sex, male (%)	38 (51)
Body mass index (kg/m^2^)	25.5 (23–28)
Type of stroke (%)	
Ischemic	58 (82)
Haemorrhagic	11 (15)
Subarachnoid	2 (3)
Hemiparetic side (%)	
Left	33 (46)
Right	22 (31)
Both sides	2 (3)
Other	14 (20)
Time since stroke (days)	16 (12–25)
Length of stay (days)	35 (19–60)
Barthel Index	12±4.6
Berg Balance scale	31 (12–46)
Trunk Control Test	100 (75–100)
Functional Ambulation Classification (%)	
Non-ambulatory (FAC 0)	25 (35)
Dependent (FAC 1–3)	27 (38)
Independent (FAC 4–5)	19 (27)
USER-mobility baseline	17±9.4
USER-mobility discharge	29 (24–33)
USER Delta mobility	11±7.2

USER: Utrecht Scale for Evaluation of Rehabilitation, IQR: interquartile range, FAC: Functional Ambulation Classification

### Selection of sway features by principal component analysis

For the PCA, 12 out of 35 postural sway features were selected based on demonstrated reliability across all IMU balance tasks. The overall KMO of each condition exceeded 0.5, indicating the suitability of conducting the PCA. The PCA including all conditions resulted in 2 principal components with eigenvalues greater than 1. For each task, more than 80% of the variance was captured in the 2 principal components. All principal components were measured with good-excellent reliability (ICC > 0.7).

### Predictive performance

For the predictive modelling, patients with a maximum (optimal) score for USER-mobility at admission (*n* = 3) or who were unable to perform balance condition 2 (*n* = 12) were excluded. The results of the 3 regression models are presented in Table II. In the linear regression analyses, the components of the balance conditions alone did not demonstrate significant contributions in Model 2 (*p* > 0.05). In the final model, which also included the USER-mobility score at admission as an independent variable, both balance conditions showed a significant contribution. The incorporation of the principal components of the balance condition in the final model led to an increased explained variance compared to model 1, where only the USER-mobility at admission was included as an independent variable (*R*^2^ = 0.61 vs 0.30).

**Table II:** Predictive performance of Utrecht Scale for Evaluation of Rehabilitation – mobility (USER-M) and balance conditions on functional recovery at discharge

Model	Dependent variable	*R* ^2^ *	*d*	Independent variable	*B*	*β*	*T*	*p*	VIF
USER-M	USER-DELTA	0.30	1.77	(Constant)	18.54				
				USER-M	–0.45	–0.59	–5.27	0.00	1.00
Balance conditions	USER-DELTA	–0.04	0.96	(Constant)	11.08				
				B1– PC 1	–0.16	–0.05	–0.30	0.77	1.33
				B2– PC 1	0.01	0.00	–0.03	0.99	1.33
USER-M + Balance conditions	USER-DELTA	0.61	2.17	(Constant)	25.04				
				USER-M	–0.80	–0.87	–8.93	0.00	1.21
				B1– PC 1	0.87	0.26	2.43	0.02	1.45
				B2– PC 1	–1.81	–0.43	–3.81	0.00	1.59

* adjusted, *d*: Durbin-Watson, *B*: unstandardized beta, *β*: standardized beta, *T*: the *t* test statistic), B1– B2: Balance condition 1 and 2, PC: Principal component, USER-DELTA: USER-mobility score at discharge minus USER-mobility score at admission; VIF: variance inflation factor

### Comparison between subgroups

Characteristics of the additional post-hoc subgroup analyses are described in Table III. The results of the 3 regression models, per subgroup, are presented in Table IV. For the regression analyses only 13 patients could be included in subgroup 1, as only 13 out of 25 were able to complete balance condition 2. In the first model, the USER-mobility score at admission alone did not demonstrate significant contributions in the FAC: 0 subgroup (*p* > 0.05). Similarly, for all subgroups the components of the balance conditions alone did not demonstrate significant contributions in Model 2 (*p* > 0.05). In the final model, the combination of principal components with USER-mobility at admission led to an increased explained variance compared to the first model for subgroups FAC: 1–3 (*R* ^2^ = 0.63 vs 0.58) and FAC: 4–5 (*R* ^2^ = 0.47 vs 0.34).

**Table III:** Characteristics of subgroups based on level of mobility on admission (Mean ±, Median [IQR])

	Not ambulatory (*n* = 25)	Require assistance (*n* = 27)	Mobilise independently (*n* = 19)
Age (years)	77.3±9.1	76.3±11.3	79.1±8.1
Sex, male (%)	12 (48%)	16 (59%)	9 (47%)
Barthel Index	7.71±4.1	13.76±3.4	14.1±3.4
Berg Balance scale	8.0 (3.5–29.0)	44.0 (31.0–48.0)	45.0 (38.0–81.0)
Trunk Control Test	75 (55–100)	100 (87–100)	100 (100–100)
USER-mobility baseline	7.80±5.5	18.21±7.1	24.31±5.3
USER-mobility discharge	23 (15.8–30.5)	30 (27.0–33.8)	33.5 (30.3–35.0)
USER Delta mobility	14.25±7.2	11.48±6.9	7.31±5.2
Length of stay (days)	53.0 (35.0–92.0)	33.5 (17.5–50.0)	20.0 (9.3–27.8)
Completed balance assessment			
Balance condition 1 (N(%))	20 (80)	26 (96)	19 (100)
Balance condition 2	13 (52)	26 (96)	19 (100)
B1–PC 1	1.34	0.35	0.14
B2–PC 1	0.6	–0.16	–0.93

Groups: not ambulatory (FAC 0), require assistance (FAC 1–3), mobilize independently (FAC 4–5), USER-M: Utrecht Scale for Evaluation of Rehabilitation – mobility, USER-DELTA: USER-mobility score at discharge minus USER-mobility score at admission. B1– B2: Balance condition 1 and 2, PC: Principal component B1–PC 1, B2–PC 1: lower scores indicate less sway and better balance

**Table IV:** Predictive performance per subgroup of USER-M and balance conditions

Model	Dependent variable	Group	*R* ^2*^	*d*	Independent variable	*B*	*β*	*T*	*p*	VIF
USER-M	USER-DELTA				(Constant)	15.23				
		Not ambulatory	–0.35	1.77	USER-M	–0.13	0.27	0.46	0.65	1.0
					(Constant)	26.46				
		Require assistance	0.58	2.47	USER-M	–0.84	–0.77	–5.64	0.00	1.0
					(Constant)	25.13				
		Mobilize independently	0.34	2.76	USER-M	–0.73	–0.62	–2.97	0.01	1.0
Balance conditions	USER-DELTA				(Constant)	15.32				
		Not ambulatory	0.09	1.16	B1–PC 1	1.32	0.32	1.03	0.32	1.07
					B2–PC 1	–1.38	-0.39	–1.25	0.24	1.07
					(Constant)	11.80				
		Requires assistance	–0.29	1.81	B1–PC 1	–0.08	–0.03	–0.10	0.92	1.71
					B2–PC 1	–1.05	–0.24	–0.24	0.41	1.71
					(Constant)	6.01				
		Mobilize independently	–0.50	1.37	B1–PC 1	1.11	0.39	1.13	0.28	1.67
					B2–PC 1	–0.97	–0.23	–0.66	0.52	1.67
USER-M + Balance conditions	USER-DELTA				(Constant)	21.95				
					USER-M	–0.57	–0.39	–1.25	0.25	1.31
		Not ambulatory	0.07	1.71	B1–PC 1	1.16	–0.28	0.93	0.38	1.09
					B2–PC 1	–1.79	–0.51	–1.61	0.15	1.18
					(Constant)	26.14				
					USER-M	–0.83	–0.80	–5.84	0.00	1.04
		Require assistance	0.63	2.93	B1–PC 1	0.47	0.17	.93	0.37	1.77
					B2–PC 1	–1.40	–0.32	v1.85	0.08	1.72
					(Constant)	25.87				
					USER-M	–0.87	–0.74	–3.71	0.00	1.1
		Mobilize independently	0.47	2.45	B1–PC 1	1.50	0.52	2.13	0.06	1.71
					B2–PC 1	–2.26	–0.53	–2.07	0.06	1.86

* adjusted, *d*: Durbin-Watson, *B:* unstandardized beta, *β:* standardized beta, *T*: the *t* test statistic), B1– B2: Balance condition 1 and 2, PC: Principal component, USER-M: Utrecht Scale for Evaluation of Rehabilitation – mobility, USER-DELTA: USER-mobility score at discharge minus USER-mobility score at admission, Groups: not ambulatory (FAC 0), require assistance (FAC 1–3), mobilize independently (FAC 4–5); VIF: variance inflation factor.

## DISCUSSION

### Principal findings

In this study we investigated whether an IMU, when combined with USER, can improve the prediction of functional recovery in older adults with stroke in GR. Our 2 main findings were: (*i*) combining sitting and standing balance as measured by an IMU with USER data improves the prediction of functional recovery at discharge compared to USER alone; (*ii*) use of IMU data was not possible for non- ambulatory patients (FAC = 0).

### Comparison with previous studies

A distinctive feature of this study was the integration of assessments made across different ICF domains. Our results demonstrate that combining sitting and standing balance as measured by an IMU (ICF domain: body functions & structures) with USER data (ICF domain: activities) improves the prediction of functional recovery. These results are in line with previous studies that examined prediction of rehabilitation outcomes using technology-derived data (33–35). For instance, O’Brien et al. (33) utilised data from an IMU obtained during a brief bout of walking at admission and found that it improved the prediction of discharge walking ability in post-stroke rehabilitation. Similarly, Sprint et al. (35) investigated the use of IMU data during ambulatory tasks to predict clinical outcomes of functional independence at discharge as measured by the FIM. The performance of predictive models improved when incorporating data from multiple measurements (35), and when clinical scale data were combined with data derived from an IMU (33). However, as these studies used different clinical scales, different algorithm models, different motor functions, and different prediction models, comparison of results is difficult.

In our study a prediction model that only included balance conditions as assessed by the IMU did not yield a statistically significant prediction of the delta USER at discharge. This lack of significance may be due to the distinct constructs assessed by the IMU and the USER; specifically, the IMU assesses body structures and functions, whereas the USER focusses on activities. Zarrifa et al. (36) reported comparable findings, where certain measured constructs acquired through upper limb robotics were deemed less critical for predicting functional abilities as evaluated by clinical scales. The measured construct likely had minimal impact on functionality as defined by the clinical scale assessing functional recovery.

In our post-hoc subgroup analysis, our findings specifically indicate a higher accuracy in predicting functional recovery after stroke for 2 subgroups: patients requiring assistance with mobilisation (FAC: 1–3) and patients who could mobilise independently (FAC 4–5). Conversely, none of the models applied to the non-ambulatory subgroup of participants (FAC score: 0) produced a statistically significant prediction of the delta USER at discharge. This difference in results is presumably a result of the low number of patients in the non-ambulatory subgroup who were capable of completing balance condition 2 (52%). Moreover, the balance scores for the non-ambulatory group (FAC score: 0) are likely very homogeneous compared to the other 2 groups (FAC 1–3 and FAC 4–5), with the insufficient variation in the dependent variable explaining why it did not significantly predict the USER delta.

To the best of our knowledge, this study is the first attempt to generate insights into the usefulness of IMU-dependent balance condition assessment for improving the prediction of functional recovery after stroke in GR within specific subgroups. This study contributes not only to understanding issues related to the accuracy of predicting functional recovery but also provides valuable information regarding the feasibility of conducting balance condition assessments using an IMU.

This study had several strengths. Regarding the IMU, we used a rigorous data collection method to obtain objective, accurate, and reliable assessments of sitting and standing balance, providing comprehensive insights into balance conditions. Additionally, the inclusion of post-hoc subgroup analyses contributed to a nuanced understanding of the main findings and offered valuable insights into feasibility. Our findings suggest that the challenge level of balance measurements should align with the individual patient’s capabilities. It is crucial to ensure that the balance assessment is not excessively difficult, preventing patients from successfully completing the measurement.

However, we also acknowledge certain limitations of the study. The relatively small sample size may limit generalisability of the results to a broader population of older individuals recovering from stroke in GR. This limitation is particularly relevant for a subset of non-ambulatory patients (FAC = 0), who were unable to complete balance condition 2 and were therefore excluded from the predictive modelling analysis. Consequently, our findings from the predictive modelling analysis do not apply to this subgroup. Since the use of an IMU requires a minimum level of physical performance from the participant, utilising IMU data to predict functional recovery appears less feasible for non-ambulatory patients. Furthermore, while the results from the post hoc subgroup analyses were promising, the number of patients per subgroup was small. Lastly, while the incorporation of IMU data in the final model led to an increased the explained variance compared to a model that included only the USER-mobility score at admission, it is crucial for future studies to assess its clinical relevance, preferably by validating of these prediction models with a larger sample size.

By integrating technology-driven data with clinical scales, insights can be gained across multiple ICF domains, enabling a comprehensive understanding of each patient’s unique digital phenotype (20) and motor phenotype (37). This integration opens avenues for “precision rehabilitation” (38) facilitating the design of tailored rehabilitation interventions aligned with the patient’s capacity, potentially increasing the likelihood of an individual or subgroup responding more effectively to specific treatments (39).

In conclusion, complementing clinical scales with technology-derived data can improve the prediction of functional recovery in older adults recovering from stroke during GR. This approach appears less feasible for non-ambulatory patients. Future research should prioritise the validation of these prediction models, preferably using a larger sample size. This will enable more precise assessment of IMU-determined balance conditions, particularly within specific subgroups.

### Clinical messages

Complementing clinical scales with technology-derived data improves the prediction of functional recovery in older adults recovering from stroke

The difficulty level of IMU-based balance measurements should align with an individual patient’s capabilities

Defining a patient’s unique digital phenotype using technology-derived data will ultimately help to provide personalised rehabilitation treatment
